# Elements of Pathological Anatomy

**Published:** 1857-09

**Authors:** 


ART. VIII.—*Elements of Pathological Anatomy.*—By SAMUEL D. GROSS, M.D., Professor of Surgery in the Jefferson Medical College of Philadelphia, and formerly Professor of Pathological Anatomy in the Medical Department of the Cincinnati College. Third edition, modified and thoroughly revised. Illustrated by three hundred and forty-two engravings on wood. Philadelphia: Blanchard & Lea. 1857. Pp. 771.

IT must be a subject for grave reflection that, while the press in this country is so prolific of systematic works, both original and reprints, on the Anatomy of the healthy organs, Materia Medica and Therapeutics, Practice of Medicine, Surgery, and Obstetrics, the department of Pathological Anatomy, which has such close and inseparable connection with all the branches of practical medicine, and which is indispensable for their elucidation, should be neglected in nearly all the medical schools, and, of course, by nearly all the students of medicine, in the United States. Under circumstances so discouraging every lover of progress in our science, which implies of necessity intentness in securing sound principles for a foundation, must hail with pleasure the appearance of a new edition of Dr. Gross's Elements of Pathological Anatomy. Were it merely a rallying point for scattered observations by others,—a suggestion to systematized efforts in other quarters, this work ought to command our respectful attention. But it comes before us with higher claims than these, by its not only indicating the path of inquiry to be pursued, but, also, by a large amount of positive knowledge, available for guidance, where, wanting its aid, confusion and uncertainty would have prevailed.

We read in the preface: "All that related to histology and diagnosis in the preceding edition, has been omitted in this, many paragraphs and often whole pages have been rewritten; and a considerable amount of new matter has been introduced." One hundred and thirty new cuts have been added. In taking a view of this work, it should be borne in mind, that the author does not offer an entire system, but only elements of pathology; and hence we cannot expect pathological histology to be treated of in detail, as has been done by Wedl, under the modest title of "Rudiments," nor with the fulness in some parts, which we find in "Vogel's Pathological Anatomy of the Human Body." But this last writer could only promise to reach the consideration of pathological changes affecting special organs in a second volume, which, we believe, has not, after the lapse of some years, yet appeared. We shall not stop, at this time, to inquire into the merits of the plan pursued by Dr. Gross, nor to ask whether a description of morbid changes of tissues common to different organs ought not to precede any analytical examination of the alterations in the composite structure of such organ separately. The division of his subject adopted by the author, is based on physiology rather than on general anatomy—and, so far, it is open to criticism; but it has this great and redeeming advantage, that the attention of the reader is at once fixed, and he becomes interested in learning the morbid textural alterations which constitute the pathological anatomy of the organ whose diseases he may be called on to treat; but which, as part of a general system, distributed through the body, he would only have looked at in an abstract manner.

It would be wrong for us, however, to create a belief in the minds of our readers, that the author enters at once on a study of the lesions of the organs, or of Special Pathological Anatomy, without a preliminary notice of the morbid changes common to most of the tissues and organic systems of the body. This he gives under the head of "General Principles of Pathological Anatomy," on which occasion, he treats, in successive chapters, of inflammation, effusion of serum, lymphization, suppuration, hemorrhage, softening, gangrene, ulceration, cicatrization, induration, hypertrophy, atrophy, pustules, transformations, pneumatoses, or collections of aeriform fluids, polypes, hydatids, serous cysts, heterologous formations, which last include tubercle, melanoses, scirrhus, encephaloid, colloid, and epithelial cancer. The part performed by inflammation in alterations of structure may be readily inferred from the author's declaring, "as a general proposition liable to few exceptions; that all organic diseases, whatever be their seat or extent, are the result of inflammatory action, either of an acute or chronic kind." He had just before told us of the latitude of interpretation which he gives to the term "organic," in pathological anatomy, as expressing not only some permanent change in the textures of an organ, "but, also, every temporary alteration which the tissues experience when in a state of disease."

In referring to the second part of the volume, devoted to special pathological anatomy, we might find occasion to criticise the arrangement adopted by the author. It seems to us, that the first chapter, or that on the blood, might well have formed a part of general pathological anatomy, and have preceded that on inflammation, the phenomena and terminations of which consist so much in the changes and conversions of this almost omnipresent fluid in the animal organism. The same remark applies, to a considerable extent, to the chapter on the cellular texture, which is the matrix of so many of the organic systems and a constituent of nearly all the organs. More to the purpose, however, than the feature of arrangement, is a lucid and succinct description of the morbid changes in the blood and the cellular tissue, which are found in the present volume. To these topics succeed, in the following order, chapters on the adipose texture, muscular system, arteries, veins, lymphatic vessels and ganglions, joints, osseous system, cutaneous system, nervous system, eye, ear, thymus gland, thyroid gland, respiratory apparatus, heart and its membranes, nasal, maxillary, and frontal cavities, mouth, pharynx, and

œsophagus, stomach and bowels, peritoneum, biliary apparatus, spleen, pancreas, urinary apparatus, male organs of generation, female organs of generation.

The numerous wood-cuts, two hundred of which were drawn from the author's own specimens, facilitate greatly a clear understanding of the text, while they show, at the same time, the large amount of original material in the preparation of the present work.

Teachers and writers on the practice of medicine and on surgery, as well as on obstetrics, may, severally, lay claim to large portions of this volume, as belonging to their branches; whereas, its contents are and ought to be recognized as common property for the use and benefit of all; or, changing the comparison, its contents constitute the necessary foundation of their different superstructures, which wanting it must be flimsy, and indifferently calculated to fulfil the original design of their framers. But of this more anon. In the mean time we offer our thanks and cordial greetings to Dr. Gross, for helping to lay a good and broad foundation. His forthcoming work on surgery will, we venture to say, show how highly he estimates the value of such a support.

